# Structure-Aware Joint Sparse Optimization for Point Cloud Denoising

**DOI:** 10.3390/s26154749

**Published:** 2026-07-26

**Authors:** Bibo Zhang, Min Wang

**Affiliations:** Ocean College, Jiangsu University of Science and Technology, Zhenjiang 212100, China

**Keywords:** point cloud, denoising, joint sparse optimization, group-level sparsity, structural integrity

## Abstract

Point clouds are widely used to represent real-world objects in the fields of augmented/virtual reality, robotics, etc. However, they are often corrupted with noise, hindering downstream tasks such as mesh surface reconstruction, rendering, etc. In this paper, we revisit sparse point cloud representation for denoising. We observe that existing works generally assume global sparsity of noise across point cloud surfaces, leading to over-smoothing, and typically adopt multiple stages to recover features, inevitably introducing biases and structural inconsistencies. To address these challenges, we propose a structure-aware joint sparse optimization approach for point cloud denoising. Specifically, considering that sparsity primarily lies in feature areas, we propose a novel group-level sparsity-based approximate deviation to characterize surface distortion for different structures. Based on that, we develop an optimization model that can protect structural integrity. We further derive a linear approximate solution and provide a parallel denoising algorithm. Experimental results on different types of datasets demonstrate that the proposed approach outperforms the state-of-the-art works.

## 1. Introduction

A point cloud is a set of data points in 3D space representing the shape of an object or scene, which is typically captured by sensing devices (e.g., LIDARs, 3D scanners, etc.) or generated by reconstruction algorithms, such as multi-view stereo. Point clouds have recently been gaining increasing momentum in industry due to their capability of providing a solid geometric representation of real-world objects, which can be applied in a variety of emerging fields, such as autonomous driving, robotics, computer-aided geometric design, etc. However, such applications are inhibited by the fact that point clouds are perturbed with a non-negligible amount of noise during acquisition [1], affecting downstream tasks, including mesh surface reconstruction and rendering [2], point cloud completion [3,4], etc. This necessitates effective and high-fidelity 3D shape restoration from noisy point clouds.

Recent years have seen research carried out to recover noise-free point clouds. Several approaches [5,6,7,8,9] project noisy points to the underlying surfaces based on moving least squares, assuming piecewise smooth surface priors. They tend to blur geometric features to varying degrees. Some other works [10,11,12,13,14,15] generate point clouds with uniform point distributions for denoising based on the concept of particles but may produce gaps near features in point clouds. Recently, much attention has been paid to data-driven methods, especially learning-based ones [16,17,18,19,20,21]. These approaches effectively remove noise and preserve geometric features but lack interpretability, have limited generalization capacity, and require intensive training. There are a few other works falling into the category of sparse representation, which was initially adopted in image processing and subsequently introduced in point cloud denoising. The existing sparse optimization-based approaches [22,23,24,25] leverage the sparsity of noise information for denoising and can yield impressive results but require multi-stage processing and thus cause structural inconsistency near features.

In this paper, we revisit sparse optimization-based point cloud denoising with the following observations. Although sparse optimization has demonstrated prospects in point cloud denoising, the existing methods typically assume global sparsity of noise across the entire point surface, which can lead to over-smoothing. Meanwhile, they typically process point clouds via multiple stages, including preliminary surface smoothing and post-feature recovery, introducing structural incoherence and biases into a point cloud and failing to protect structural integrity.

**Figure 1:**
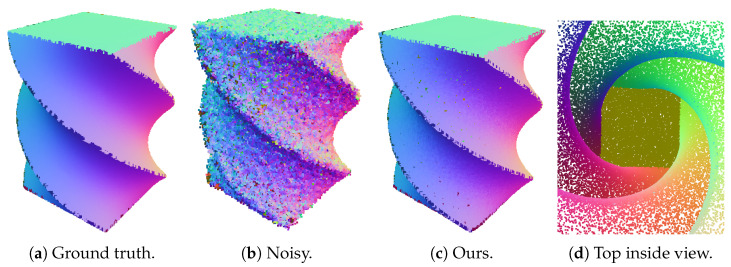
Denoising results of the proposed approach on a twisted vase point cloud (88,518 points; edge angles <30°). (**a**) is the ground truth. (**b**) is the input model with Gaussian noise (standard deviation: 2% of the bounding box diagonal length). (**c**) is the point model processed by the proposed approach. (**d**) is the top inside view of the model.

Motivated by the above observations, we propose a structure-aware joint sparse optimization approach for point cloud denoising based on the fact that sparsity is typically confined to specific features in practice. In particular, we introduce the concept of latent groups and identify a noisy point as a *facet* or *feature* point accordingly. Then, a unified group-level sparsity-based distance measure applicable to both types of points is introduced, which captures the distance of a noisy point from latent groups rather than individual neighbor points. Based on that, we propose an $\ell_{0}$ optimization model and obtain a linear approximate solution, which allows parallel repositioning for all points. A representative result of our approach can be found in Figure 1. The main contributions of this paper are summarized as follows.

- We propose a latent group and feature identification approach and design a novel group-level sparsity-based measure to capture geometric characteristics of different structures.
- We build a structure-aware $\ell_{0}$ sparse optimization model that is capable of jointly restoring both feature and facet points, thus preserving the structural integrity of point clouds and avoiding biases and inconsistencies.
- We derive a linear alternating approximate solution to the optimization model and provide a parallel denoising algorithm.
- We evaluate the performance through extensive experiments on synthetic point clouds and real-world raw scans and demonstrate that it can outperform the baselines.

The rest of the paper is organized as follows. Section 2 categorizes and reviews the existing related works. In Section 3, the primary details of the proposed approach are presented. Then, we present a parallel denoising algorithm in Section 4. The results of the numerical evaluation are showcased and discussed in Section 5. Finally, Section 6 concludes the paper with some final remarks.

## 2. Related Work

Point cloud denoising works mainly fall into four categories: moving least squares (MLS)-based, particle-based, learning-based, and sparsity-based methods.

**MLS-based methods.** The MLS approach aims to build a smooth meshless surface approximation of a noisy point cloud. Several extensions of it have been widely adopted to process point clouds. The point set surface (PSS) approach [5] defines a smooth surface by locally approximating the raw point cloud using the MLS method. Shen et al. [6] propose an implicit MLS (IMLS) surface representation that defines a weighted average of tangential implicit planes associated with noisy samples. Guennebaud et al. [7] present an algebraic point set surface (APSS) approach to reconstruct surfaces via projection to algebraic spheres based on MLS. In [8], Öztireli et al. propose robust IMLS (RIMLS) by combining IMLS and local kernel regression to reconstruct point set surfaces. Xu et al. [9] jointly optimize point positions and normals based on a generalized robust error metric. MLS-based methods iteratively project noisy points onto an approximated underlying surface that is assumed to have piecewise smooth priors. This inevitably blurs geometric features, especially sharp ones, to varying degrees.

**Particle-based methods.** Particles are uniformly distributed points that represent the underlying surface shape of a raw point cloud [26]. The locally optimal projection (LOP) [10] performs a second-order approximation to smooth surfaces without the need for any explicit or implicit approximation space. The weighted LOP (WLOP) [11] produces noise-free particles and presents an iterative predictor–corrector scheme for robust normal estimation. The continuous LOP (CLOP) [12] enhances the computational efficiency of the WLOP by introducing a continuous representation for point sets based on Gaussian mixture models. Edge-aware resampling (EAR) [13], based on WLOP, performs both normal estimation and resampling progressively from surfaces to edges. Liu et al. [14] present an anisotropic second-order regularization-based normal restoration method and a RANSAC-based point updating approach. Chen et al. [15] incorporate, in energy minimization, a repulsion term for even point distribution and a data term for latent surface approximation. Particle-based methods produce uniformly distributed sampling points but can generate gaps near features in point clouds.

**Learning-based methods.** Following the momentum that learning-based methods have gained in the field of vision, deep learning-based point cloud processing has been attracting attention. PointNet [16] was first proposed in shape classification and segmentation, which processes points independently before global aggregation. A few approaches extend PointNet to point cloud denoising. Rakotosaona et al. propose PointCleanNet [17] to estimate correction vectors that project noisy points onto clean surfaces. Roveri et al. present PointProNet [18], which employs a deep architecture similar to PointNet to estimate local surface directions. Pistilli et al. [19] propose a graph convolutional neural network-based point cloud denoising and outlier removal approach. Wang et al. [20] introduce an unsupervised learning-based point cloud denoising method, which does not require ground-truth point clouds during training. In [21], the authors present a 3D patch-based contrastive learning framework to pre-train an encoder and construct a joint-loss regression network for simultaneous point cloud denoising and normal estimation. Despite the advantages of neural networks, learning-based methods lack interpretability and may face generalization issues. Addressing this typically requires hyperparameter tuning and re-training on new datasets, which are both time-consuming and inconvenient. Additionally, these approaches rely heavily on compute-intensive hardware, such as GPUs, which limits the applicability of the approaches.

**Sparsity-based methods.** Sparse representation was initially introduced into image processing [27,28], demonstrating its potential in dealing with visual tasks. Xu et al. [29,30] apply $\ell_{0}$ optimization to image smoothing and motion deblurring. Also, sparsity-based mesh surface approximation approaches have emerged. He et al. [31] apply $\ell_{0}$ optimization to 3D mesh model denoising. Wang et al. [32] use global Laplacian regularization to estimate mesh models and recover sharp features through $\ell_{1}$ compressed sensing optimization. Xiong et al. [33] formulate surface reconstruction as a constrained $\ell_{2,q}$ optimization (0<q<1) problem and solve it by a dictionary learning approach. Zhang et al. [34] propose a variational mesh denoising method by combining piecewise constant function spaces with the total variation. Wang et al. [35] construct manifolds from discrete meshes through sparse optimization.

Meshless models like point clouds lack point connectivity that implicitly defines surface topology, introducing new challenges for denoising. Avron et al. [24] reconstruct piecewise smooth point set surfaces by adopting $\ell_{1}$ representations on both normals and point positions. Mattei et al. [25] use local averages to estimate point positions and preserve sharp features through weighted $\ell_{1}$ minimization. Wang et al. [22] smooth volume point sets via $\ell_{0}$ gradient minimization with a post-processing procedure to adjust erroneous points near or on boundaries. Sun et al. [23] propose a multi-stage $\ell_{0}$-based denoising approach that first smooths a point cloud over the whole surface and then recovers edges.

The existing sparsity-based approaches assume global sparsity of noise and adopt multiple point processing stages, i.e., preliminary smoothing for all points and post-recovery for feature points. However, such separate stages can lead to structural incoherence and biases in a point cloud, thus affecting structural integrity, such as the gaps and holes shown in Section 5. Additionally, they are sensitive to noise levels and normal estimation accuracy.

In this paper, we revisit sparse optimization, particularly in the $\ell_{0}$ norm, for point cloud denoising. Different from the above existing works, we propose a structure-aware $\ell_{0}$ optimization model to jointly denoise different surface areas based on the observation that sparsity primarily exists in feature regions. We further derive a linear approximate solution with a parallel denoising implementation.

## 3. Structure-Aware Joint Sparse Optimization Approach

In this section, we propose a structure-aware joint sparse optimization approach for point cloud denoising. We first briefly introduce the normal estimation and smoothing scheme and then design a latent group and feature identification approach for local structure representation of the point surface. Subsequently, we develop a sparse optimization model and derive a linear approximate solution that enables parallel processing for all points.

### 3.1. Overview

**Figure 2:**
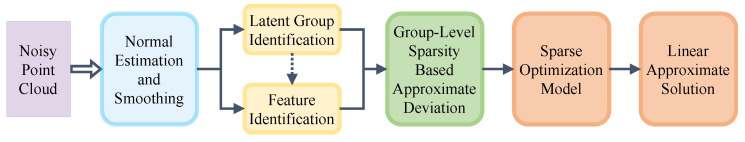
An overview of the structure-aware joint sparse optimization approach. The hollow thick arrow indicates the input, while solid thin arrows show the dependencies among the components.

The approach presented in this section underpins the parallel denoising algorithm developed in the next section, and Figure 2 provides a high-level overview. As the initial step, the normal estimation and smoothing scheme computes and smooths point normals, which are subsequently fed, together with the point positions, to the downstream components. The latent group and feature identification partitions the neighborhood surface of a point into groups corresponding to locally flat regions and further identifies features, such as edges. Relying on these neighborhood characteristics, we compute the group-level sparsity-based approximate deviation for each point, which is fundamental to the optimization model constructed thereafter. Subsequently, a sparse optimization model is built to denoise the point cloud while ensuring fidelity via surface smoothing and feature preservation. Finally, a linear approximate solution of the optimization model is derived, facilitating parallel point processing.

### 3.2. Normal Estimation and Smoothing

We use local plane fitting methods, e.g., principal component analysis (PCA), to estimate normals for point clouds. However, it potentially ignores varying surface structures. Post-smoothing methods are often employed to mitigate this issue. The general neighborhood averaging method may over-smooth sharp features, while sparse optimization methods may over-sharpen features. To preserve features, we smooth normals using bilateral filtering [8,13], a weighted averaging method based on Gaussian distribution. Specifically, let ${\left\{p_{i}\right\}}_{i\in I}$ and ${\left\{n_{i}\right\}}_{i\in I}$ denote the raw point cloud and the corresponding normal set, where I is the point index set and $p_{i}$ is the *i*-th point’s position. Then, the normals are smoothed through following updates:

(1) $$n_{i}:=\frac{\sum_{j\in N_{i}}\phi (∥p_{i}-p_{j}{∥}_{2})\psi \left(n_{i}^{T}n_{j}\right)n_{j}}{\sum_{j\in N_{i}}\phi (∥p_{i}-p_{j}{∥}_{2})\psi \left(n_{i}^{T}n_{j}\right)},\;\forall i\in I,$$

where $N_{i}$ is the index set of $p_{i}$’s neighbors in a radius of $\sigma_{p}$. $\phi \left(d\right)=e^{-d^{2}/\sigma_{p}^{2}}$ and $\psi \left(d\right)=e^{-{\left(1-d\right)}^{2}/{\left(1-\cos\left(\sigma_{n}\right)\right)}^{2}}$ are Gaussian weight functions. $\sigma_{n}$ measures the similarity of neighbor normals. Note that normal vectors are normalized in advance for simplicity.

### 3.3. Latent Group and Feature Identification

**Figure 3:**
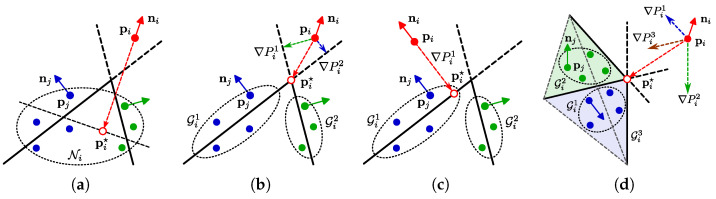
Different principles of point repositioning near features. Solid lines represent unknown ground-truth surfaces, and dotted ellipses indicate latent groups. Dashed arrows illustrate the approximate deviation, with red ones specifically indicating point repositioning. The red point $p_{i}$ is repositioned to $p_{i}^{*}$ in different cases. The green and blue points are $p_{i}$’s neighbors in different latent groups. Via (**a**) local plane projection (in *existing works*), $p_{i}$ is treated as a facet point and repositioned to $p_{i}^{*}$, which deviates from the edge. Through (**b**–**d**) multiple group-level projections (in *our approach*), $p_{i}$ is respectively identified as (**b**) edge point, (**c**) facet point and (**d**) vertex point and restored based on the corresponding group(s).

An intuitive idea of denoising is to reposition points through minimizing the distance between a noisy point and the fitting plane constructed from its neighbors. However, this may incorrectly position points near features. Figure 3a depicts a typical case, where the distance metric for an arbitrary point $p_{i}$ and its surrounding fitting plane is $\frac{1}{|N_{i}|}\sum_{j\in N_{i}}n_{i}^{T}\left(p_{i}-p_{j}\right)$. Minimizing such distance results in a new position $p_{i}^{*}$, which is not located at the intersection of true latent planes. This essentially smears edge features. Similar cases can also be seen in other types of feature areas (e.g., vertices). Moreover, this method is sensitive to normals that are often inappropriately estimated, especially in discontinuous areas (i.e., features), and further affects feature restoration.

To resolve the above issues, we categorize points into three types, namely *facet*, *edge* and *vertex* points. Facet points are those potentially lying on or close to flat and smooth surfaces; edge points are located at or close to an intersection of two facets; vertex points are those on or close to an intersection of three or more facets. We refer to both edge and vertex points as *feature* points. Further, we generate homogeneous group(s) in the neighborhood and determine the point type based on the associated groups.

**Latent group.** We divide the neighbors of a point $p_{i}$ into latent group(s) according to the normals of neighbors: $N_{i}={⋃}_{m\in M_{i}}G_{i}^{m}$, where $M_{i}=\left\{1,2,\ldots \right\}$ is the group index set and $G_{i}^{m}$ is the point index set in group $m\in M_{i}$. An underlying facet is associated with a group $G_{i}^{m}$.

**Feature identification.** Based on the group(s) that $p_{i}$ belongs to, $p_{i}$ is identified as a facet/feature point. As illustrated in Figure 3b, the neighbor points of $p_{i}$ are clustered into two groups, i.e., $G_{i}^{1}$ and $G_{i}^{2}$, and $p_{i}$ belongs to both. Therefore, $p_{i}$ is identified as an edge point and should be repositioned to the edge where two facets intersect. In Figure 3c, a facet point is expected to be placed on a facet defined by $G_{i}^{1}$. In Figure 3d, a vertex point belonging to three or more groups should be restored to the intersection tip. The detailed procedures of the above latent group and feature identification approach are further elaborated in Section 4.

### 3.4. Group-Level Sparsity-Based Approximate Deviation

We observe that sparsity mainly exists in the structural surfaces like edges, vertices, and other more complex features. The global noise sparsity assumption of the existing approach can lead to over-smoothing due to the nature of sparse optimization. This motivates us to design a group-level sparsity-based approximate deviation that can jointly characterize both soft features on facets and sharp ones at edges or vertices.

Recovering a 3D shape requires the distance(s) between a point $p_{i}$ and the associated underlying facet(s) to be minimized. Based on this principle, we define the approximate deviation of $p_{i}$ as a vector consisting of the distance(s) between $p_{i}$ and the latent facet(s), i.e., $\nabla P_{i}={\left[\nabla P_{i}^{m}\right]}_{m\in M_{i}}$, where the *m*-th element $\nabla P_{i}^{m}$ is the distance between $p_{i}$ and the *m*-th group, and defined as the average projection distance between $p_{i}$ and the neighbor points in the *m*-th group:

(2) $$\nabla P_{i}^{m}=\frac{1}{|G_{i}^{m}|}\sum_{j\in G_{i}^{m}}n_{j}^{T}\left(p_{i}-p_{j}\right),\;\forall i\in I,\;\forall m\in M_{i}.$$

Note that, in Equation (2), instead of using $n_{i}$ to compute the distance between $p_{i}$ and a *single* fitting plane estimated from all the neighbor points, as shown in Figure 3a, we project the vector $\left(p_{i}-p_{j}\right)$ onto the neighbor normals ${\left\{n_{j}\right\}}_{j\in G_{i}^{m}}$, as illustrated in Figure 3b–d, to consider *multiple* facets generated by the latent group and feature identification approach.

This newly defined approximate deviation unifies distance measures for all types of points (both feature and facet points), enabling the construction of a single optimization model applicable to both. Therefore, the denoising process can be simplified by performing restoration for all points without requiring post-processing stages (e.g., edge recovery). This can effectively preserve the structural integrity of the point cloud and avoid the biases and inconsistencies that can be introduced in multi-stage approaches. Moreover, it can facilitate the derivation of the optimization model solution and enhance the robustness of the proposed approach to unreliable normals.

### 3.5. Model and Linear Approximate Solution

#### 3.5.1. Optimization Model

Based on the approximate deviation, we propose a sparse $\ell_{0}$ optimization model for point cloud denoising as follows:

(3) $$\min_{{\left\{p_{i}\right\}}_{i\in I}}\;\sum_{i\in I}\{∥p_{i}-{\tilde{p}}_{i}{∥}_{2}^{2}\;+\lambda \sum_{m\in M_{i}}{∥\nabla P_{i}^{m}∥}_{0}\},$$

where ${\tilde{p}}_{i}$ denotes *i*-th noisy point in a raw point cloud. The first quadratic term computes the difference between the denoised point cloud and the raw one, which is used to guarantee the fidelity. The second $\ell_{0}$ regularization term is the sparsity penalty, accounting for simultaneously smoothing surfaces and protecting features. $\nabla P_{i}^{m}$ is $p_{i}$’s deviation from *m*-th facet, computed using Equation (2). Note that $∥\nabla P_{i}^{m}{∥}_{0}$ takes binary values, indicating whether $p_{i}$ lies on the *m*-th facet estimated from $G_{i}^{m}$. The significance of the second term is controlled by the coefficient λ.

Through solving the optimization problem formulated above, both facet and feature points are processed, as shown in Figure 3b–d. If $p_{i}$ is a facet point, $p_{i}$ will be repositioned to the latent facet formed by its neighbor group $G_{i}^{1}$. If $p_{i}$ is an edge point, $p_{i}$ will be restored to the edge, the intersection of associated $G_{i}^{1}$ and $G_{i}^{2}$. The restoration of vertex points with three or more associated groups follows the same rationale.

#### 3.5.2. Linear Approximate Solution

As the optimization problem (3) includes an $\ell_{0}$-norm term, the problem is non-continuous and non-differentiable. We decompose it into sub-problems by introducing auxiliary variables and solve them iteratively.

We first reformulate the original problem (3) by introducing an auxiliary variable $\omega_{i}^{m}$:

(4) $$\min_{p_{i},\omega_{i}^{m}}\sum_{i\in I}\{{∥p_{i}-{\tilde{p}}_{i}∥}_{2}^{2}+\;\;\sum_{m\in M_{i}}\;\;\{{\beta |\nabla P_{i}^{m}-\omega_{i}^{m}|}^{2}+\lambda {∥\omega_{i}^{m}∥}_{0}\}\},$$

where β controls the similarity between the auxiliary variable $\omega_{i}^{m}$ and $\nabla P_{i}^{m}$. If β→∞, $\nabla P_{i}^{m}=\omega_{i}^{m}$ is enforced and (4) becomes the same problem as (3). Through reformulation (4), we decompose the original problem into two alternating sub-problems that can be solved iteratively and finally converge to an approximate solution to (3). We defer the full derivation of solutions to Appendix A and Appendix B.

  **Sub-Problem I: Compute ${\left\{\omega_{i}^{m}\right\}}_{i\in I,m\in M_{i}}$.**

By fixing ${\left\{p_{i}\right\}}_{i\in I}$ in (4), we derive Sub-Problem I:

(5) $$\min_{\omega_{i}^{m}}\;\sum_{i\in I}\sum_{m\in M_{i}}\left\{\beta |\nabla P_{i}^{m}-\omega_{i}^{m}{|}^{2}+\lambda {∥\omega_{i}^{m}∥}_{0}\right\}.$$

As variables ${\left\{\omega_{i}^{m}\right\}}_{i\in I,m\in M_{i}}$ are ***independent***, they can be computed ***in parallel*** by simultaneously computing each $\omega_{i}^{m}$ from a simplified problem:

(6) $$\min_{\omega_{i}^{m}}\;\beta {|\nabla P_{i}^{m}-\omega_{i}^{m}|}^{2}+\lambda {∥\omega_{i}^{m}∥}_{0}.$$

Through solving simplified problem in (6), we can derive the solution to the Sub-Problem I:

(7) $$\omega_{i}^{m}=\left\{\begin{array}{cc} 0, & \mathrm{if}\;\lambda /\beta >|\nabla P_{i}^{m}{|}^{2},\\ \nabla P_{i}^{m}, & \mathrm{otherwise}, \end{array}\right.\;\forall i\in I,\forall m\in M_{i}.$$

**Sub-Problem II: Compute ${\left\{p_{i}\right\}}_{i\in I}$.**

By substituting the approximate deviation in Equation (2) into (4) and fixing ${\left\{\omega_{i}^{m}\right\}}_{i\in I,m\in M_{i}}$, we obtain Sub-Problem II, a quadratic optimization problem:

(8) $$\min_{p_{i}}\sum_{i\in I}\left\{{∥p_{i}-{\tilde{p}}_{i}∥}_{2}^{2}+\beta \;\;\;\;\sum_{m\in M_{i}}\;\;\;{\left(\frac{1}{|G_{i}^{m}|}\;\;\sum_{j\in G_{i}^{m}}\;\;n_{j}^{T}\left(p_{i}-p_{j}\right)-\omega_{i}^{m}\right)}^{2}\right\}.$$

Considering multiple variables ${\left\{p_{i}\right\}}_{i\in I}$ are intertwined in the term $\sum_{j\in G_{i}^{m}}n_{j}^{T}\left(p_{i}-p_{j}\right)$, in the iterative solution of such alternating sub-problems, we replace the variables ${\left\{p_{j}\right\}}_{j\in Ii}$ with their values computed in the previous iteration, which makes the variables ${\left\{p_{i}\right\}}_{i\in I}$ ***independent***. Therefore, letting superscript *k* denote the *k*-th iteration, we can update ${\left\{p_{i}\right\}}_{i\in I}$ ***in parallel*** by solving each $p_{i}$:

(9) $$\min_{p_{i}}{∥p_{i}-{\tilde{p}}_{i}∥}_{2}^{2}+\beta \;\sum_{m\in M_{i}}{\left(\frac{1}{|G_{i}^{m}|}\sum_{j\in G_{i}^{m}}n_{j}^{T}\left(p_{i}-p_{j}^{k}\right)-\omega_{i}^{m}\right)}^{2}.$$

Ultimately, the solution to (9) can be derived as

(10) $$\begin{array}{cc} \; & p_{i}^{k+1}=U_{i}^{-1}v_{i}, \end{array}$$

(11) $$\begin{array}{cc} \; & U_{i}=I+\beta \sum_{m\in M_{i}}{\bar{n}}_{i}^{m}{\left({\bar{n}}_{i}^{m}\right)}^{T}, \end{array}$$

(12) $$\begin{array}{cc} \; & v_{i}={\tilde{p}}_{i}+\beta \sum_{m\in M_{i}}\left(\omega_{i}^{m}+\frac{1}{|G_{i}^{m}|}\sum_{j\in G_{i}^{m}}n_{j}^{T}p_{j}^{k}\right){\bar{n}}_{i}^{m}, \end{array}$$

where ${\bar{n}}_{i}^{m}=\frac{1}{|G_{i}^{m}|}\sum_{j\in G_{i}^{m}}n_{j}$, and I is an identity matrix.

Iteratively computing ${\left\{\omega_{i}^{m}\right\}}_{i\in I,m\in M_{i}}$ in Sub-Problem I and ${\left\{p_{i}\right\}}_{i\in I}$ in Sub-Problem II, respectively, based on Equations (7) and (10), can finally converge to an approximate solution of the problem (3). Note that the above linear approximate solution enables the parallel point processing in the next section.

## 4. Parallel Denoising Algorithm

Building on the proposed optimization model and solution, this section presents the algorithm for implementing parallel point cloud denoising. In particular, Algorithm 1 provides an overview of the denoising process, while Algorithm 2 details the procedures of the latent group and feature identification approach, providing necessary group and feature information for computing alternating solutions in Algorithm 1.

In Algorithm 1, the inputs include the raw point cloud ${\left\{{\tilde{p}}_{i}\right\}}_{i\in I}$, smoothing coefficient λ, the number of neighbor points *N*, the potential maximum angle in features θ, and threshold of β, i.e., $\beta_{max}$. The number of neighbor points *N* is in principle large enough to cover local surface features. The normals ${\left\{n_{i}\right\}}_{i\in I}$ of the raw point cloud are estimated via PCA and smoothed using bilateral filtering (Equation (1)). Then, we iteratively reposition points in Lines 3–8. In particular, in each iteration, we perform latent group and feature identification detailed in Algorithm 2 to obtain the group information ${\left\{M_{i},{\left\{G_{i}^{m}\right\}}_{m\in M_{i}}\right\}}_{i\in I}$. Based on this, we update ${\left(\omega_{i}^{m}\right)}^{k+1}$ and $p_{i}^{k+1}$ for all points ${\left\{p_{i}\right\}}_{i\in I}$ ***in parallel*** using Equations (7) and (10). During iterations, β is updated through multiplying with a factor μ. Theoretically, the convergence is reached as β approaches infinity; however, we restrict β below a threshold $\beta_{max}$ to limit the processing time.
**Algorithm 1:** Parallel Point Cloud Denoising
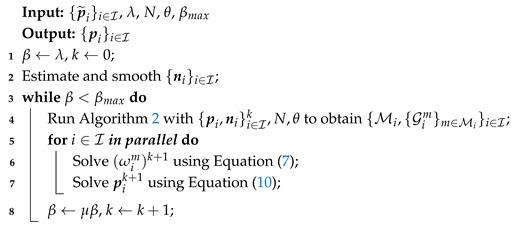


In Algorithm 2, the inputs include the raw point cloud and associated normals ${\left\{p_{i},n_{i}\right\}}_{i\in I}$, the number of neighbor points *N*, and the potential maximum angle in features θ. First of all, the nearest neighbor points for each point $p_{i}$ are collected through the KD-tree-based K-nearest neighbor (KNN) method (Line 2). Then, for each point $p_{i}$, the neighbor points are clustered into groups based on the similarity of normals (Lines 4–10). Specifically, $R_{i}$ is the index set for $p_{i}$ and its neighbors, ordered based on similarity to $p_{i}$. We use the projection distance $n_{i}^{T}n_{j}$ between two normals to measure similarity and a threshold of normal similarity cos(π−θ), i.e., −cosθ, to judge whether the examined points are in the same group. Therefore, an appropriate θ is required: a large θ makes the denoising process sensitive to noise, whereas a small θ may fail to preserve features. Each time, we pick the first element $R_{i}\left(1\right)$ in the ordered set $R_{i}$ and check the similarity between its indexed point and the other points indexed in $R_{i}$. The indices of neighbor points with sufficient similarity, examined based on θ, form a new group $G_{i}^{m}$, and its index *m* is included in the group index set $M_{i}$. The condition $|G_{i}^{m}|>1$ is to avoid the invalid group that only contains $R_{i}\left(1\right)$ itself or remains empty. Thereafter, both $R_{i}\left(1\right)$ and the point indices added to the new groups are removed from $R_{i}$. This process is repeated until $R_{i}$ is empty.
**Algorithm 2:** Latent Group and Feature Identification
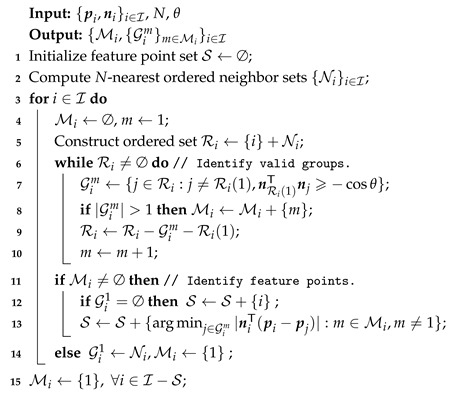


Once generating different groups, we identify the types (i.e., facet, edge and vertex) of points (Lines 11–13). Specifically, supposing groups are found (i.e., $M_{i}\neq \emptyset$), we check the size of the first group $G_{i}^{1}$. If $G_{i}^{1}$ is empty, $p_{i}$ is identified as a feature point (edge or vertex point) (Line 12) because there is no neighbor point similar to $p_{i}$ and collected into group $G_{i}^{1}$. Then, we further identify potential feature points in other groups, adopting $p_{i}$ as the reference point (Line 13). Considering that feature points are generally located on the intersections of different groups that constitute latent local facets, we intuitively locate other feature points based on the minimum absolute projection distance: for each valid group $m\in M_{i}$, a point is identified as a feature point and added to the feature point set S if it is characterized by the minimum absolute projection distance from $p_{i}$ (i.e., ${\arg\min}_{j\in G_{i}^{m}}\left|n_{i}^{T}\left(p_{i}-p_{j}\right)\right|$). A point $p_{i}$ is regarded as a facet point, and all the neighbor points $N_{i}$ constitute one group $G_{i}^{1}$ if the normals of both $p_{i}$ and its neighbors $N_{i}$ are different from each other (i.e., $M_{i}=\emptyset$) (Line 14). Note that, for a facet point $p_{i}$, the closest group $G_{i}^{1}$ is leveraged to restore $p_{i}$’s position, whereas, for a feature point $p_{i}$, all the groups indexed in $M_{i}$ are used in repositioning $p_{i}$ (Line 15).

**Complexity analysis.** The complexity of the proposed approach is determined by three parts, including (a) KD-tree-based neighbor point searching, (b) latent group and feature identification, and (c) point reposition. The complexity of part (a) is O(|I|log|I|), while the complexity for both (b) and (c) is O(N). Then, the complexity of each iteration is O(N+|I|log|I|). Finally, considering the number of iterations is approximately $\log_{\mu }\left(\beta_{max}/\lambda \right)$, the complexity of the algorithm is $O((N+\left|I|\log\left|I\right|\right)\log_{\mu }(\beta_{max}/\lambda ))$.

## 5. Experimental Results

In this section, we experiment with the proposed approach using extensive synthetic point clouds and real-world raw scans and compare it with representative baseline approaches. Both the geometric details of denoised point models and the impact of different parameters on our approach are investigated. The restoration performance is discussed first quantitatively and then visually.

### 5.1. Experimental Settings

#### 5.1.1. Datasets

**Figure 4:**
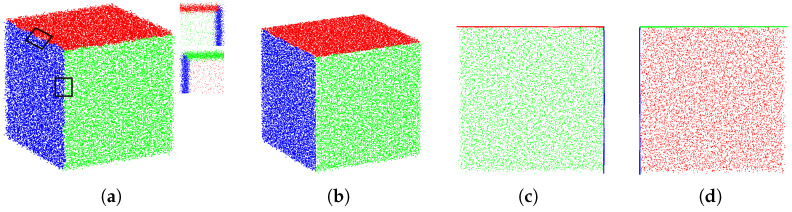
Denoising results of the proposed approach on a noisy cubic corner. (**a**) is the input noisy point cloud. (**b**–**d**) show the smoothed geometry from different views.

**Figure 5:**
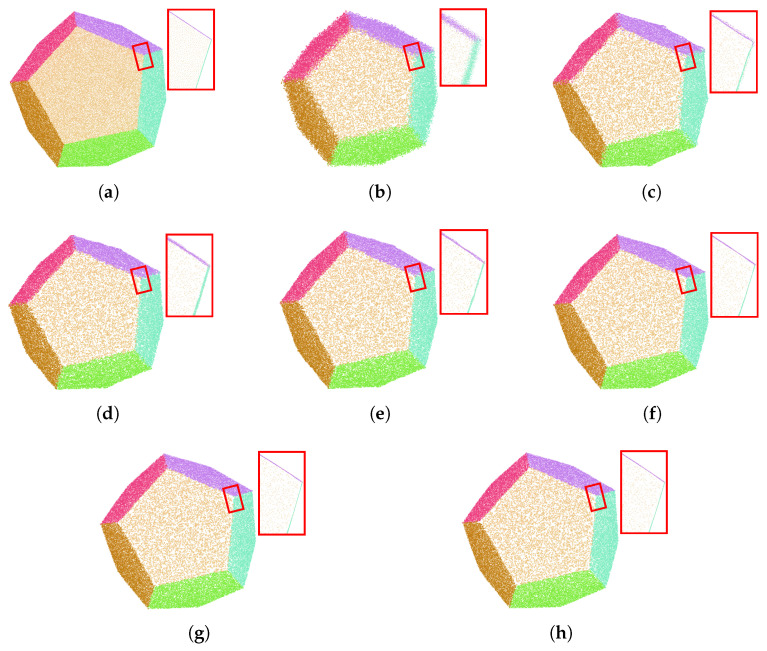
Results of the proposed approach with different parameters (the potential maximum angle in features θ, the smoothing coefficient λ, and the number of iterations iter) on a dodecahedron model (72,719 points; edge angle: 108°). (**a**) Ground truth. (**b**) Noisy. (**c**) λ=0.1,iter=8,θ<108°. (**d**) λ=0.1,iter=4,θ>108°. (**e**) λ=0.01,iter=8,θ>108°. (**f**) λ=0.1,iter=8,θ>108°. (**g**) λ=1,iter=8,θ>108°. (**h**) λ=10,iter=8,θ>108°.

**Figure 6:**
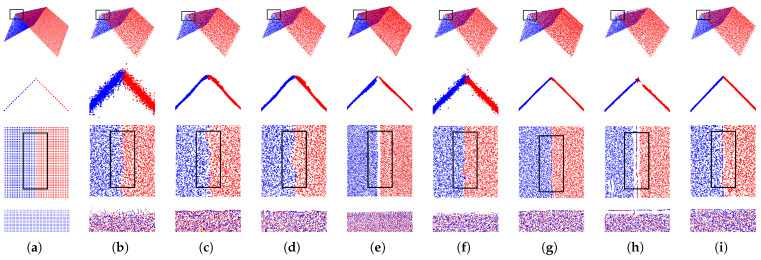
Denoising results of all approaches on a wedge point cloud. (**a**) is the ground truth. (**b**) is the input noisy point cloud. (**c**–**i**) show the outputs of different approaches. The first row shows the overall structure of the wedge. The second through fourth rows show the details from the front, top, and left views, respectively. (**a**) Ground truth. (**b**) Noisy. (**c**) APSS. (**d**) RIMLS. (**e**) EAR. (**f**) PCN. (**g**) CLJNEPCF. (**h**) LM. (**i**) Ours.

**Figure 7:**
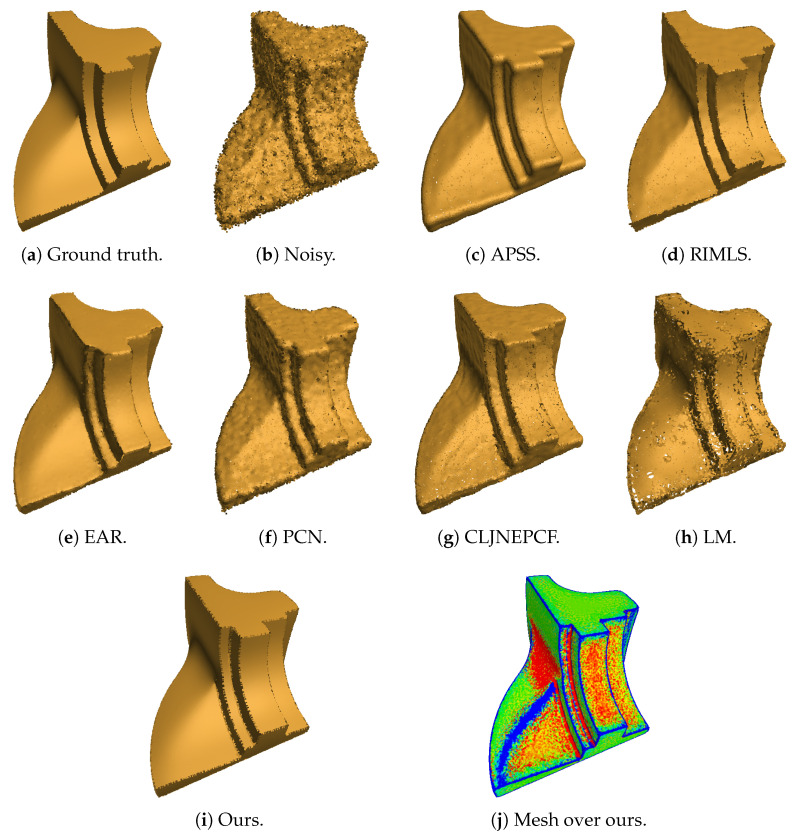
Denoising results on a Fandisk model. (**a**) is the ground truth, and (**b**) is the input noisy point cloud. (**c**–**i**) show the results of different approaches. (**j**) shows the colored mesh built on the point cloud generated by the proposed approach.

**Figure 8:**
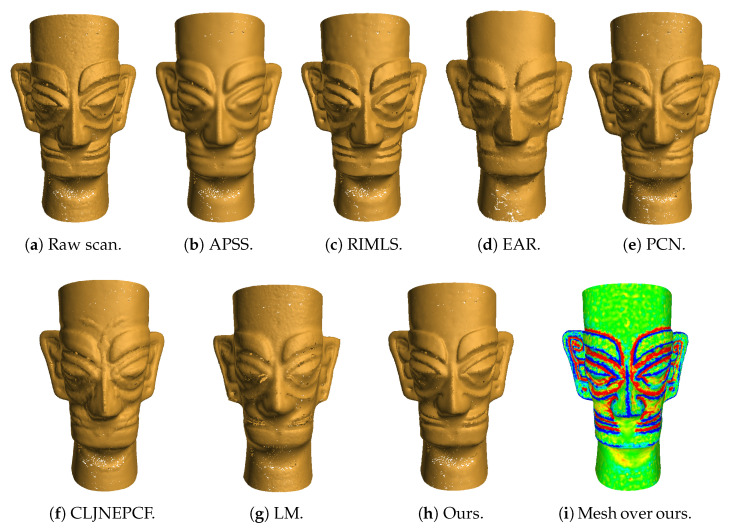
Results on the raw scan of a bronze sculpture. (**a**) is the input model. (**b**–**h**) are models processed by different approaches. (**i**) shows the colored mesh built on the point cloud processed by the proposed approach.

**Figure 9:**
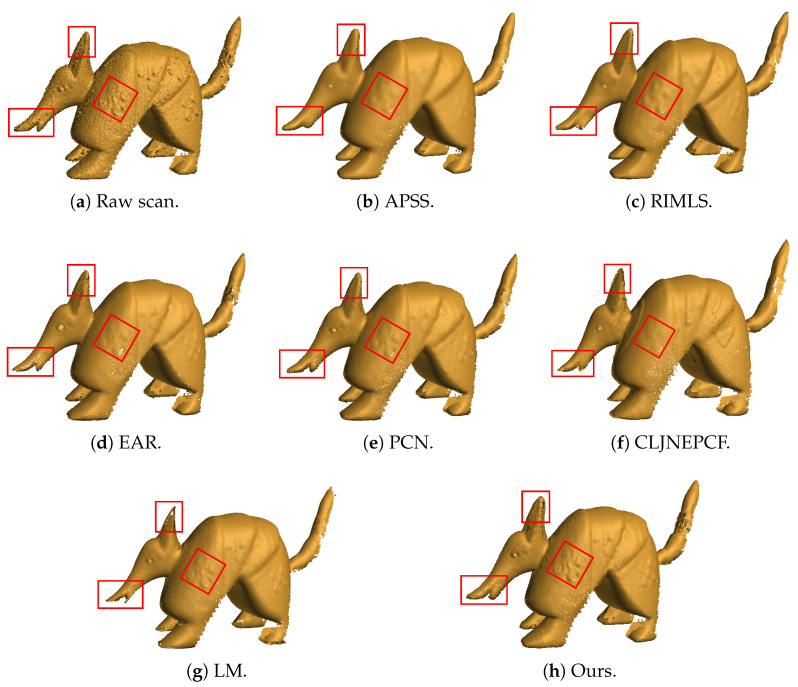
Denoising results on the raw scan of an armadillo model. (**a**) is the input model, while (**b**–**h**) present the models precessed by different approaches. The red boxes highlight the noteworthy detailed features.

**Figure 10:**
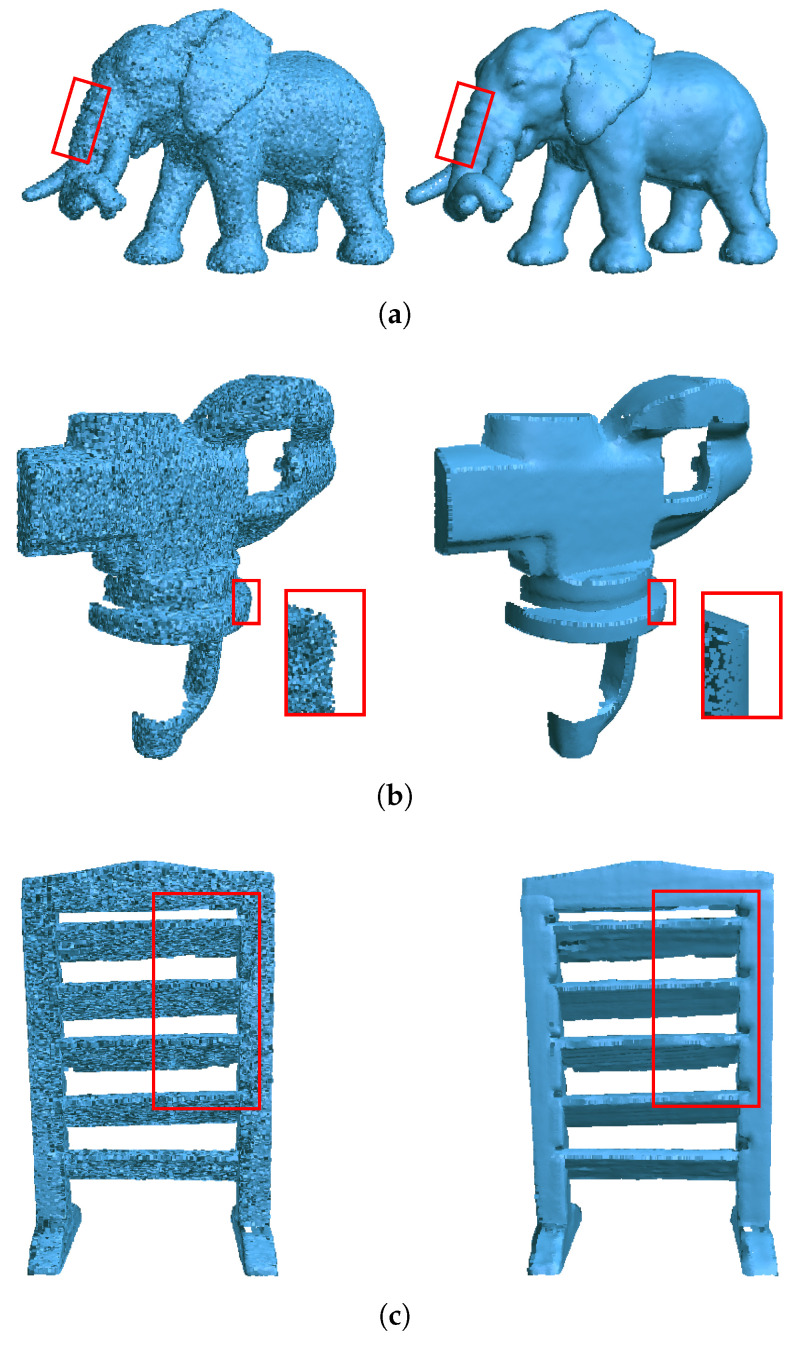
Denoising results of our approach on different raw scan models. The red boxes highlight the noteworthy detailed features. (**a**) Elephant; (**b**) Iron vise; (**c**) Shutter.

The datasets involve synthetic point clouds with both regular and complex irregular geometries and real-world raw scans with both sharp features and delicate textures (i.e., soft features). As for the synthetic point clouds, the cube, dodecahedron, and wedge (Figure 4, Figure 5, and Figure 6, respectively) serve as representative examples of different regular geometric shapes. The complex irregular geometric shapes include the twisted vase with extremely sharp edges (<30°) (Figure 1) and the Fandisk whose edge angles are approximately 90° (Figure 7). The real-world raw scans include the sculpture (Figure 8), the armadillo (Figure 9), the elephant (Figure 10a), the iron vise (Figure 10b) and the shutter (Figure 10c). Among these, the sculpture exhibits features of varying salience. The iron vise and shutter are typically characterized by sharp features, while the armadillo and elephant show a considerable amount of soft features. All the synthetic point clouds are manually corrupted with Gaussian noise with a standard deviation of 2% of the bounding box diagonal length. No extra noise is added to the real-world raw scans except the elephant point cloud that has limited noise.

#### 5.1.2. Baselines

We compare our approach with representative approaches including APSS [7], RIMLS [8], EAR [13], PointCleanNet (referred to as PCN) [17], CLJNEPCF [21] and $\ell_{0}$ minimization approach [23] (referred to as LM). The APSS and RIMLS are implemented by the MeshLab [36], and the EAR is available online https://vcc.tech/research/2013/EAR (accessed on 16 June 2026). For the PCN and CLJNEPCF, we use the denoising models and parameters provided by the authors to process the datasets. The LM is implemented according to the paper, and the main parameters are tuned as well as possible. To ensure a fair and direct comparison among approaches, no upsampling is performed on the results.

#### 5.1.3. Approach Settings

The number of neighbor points *N* depends on the point density and is set large enough to cover local surface features, whose typical values are set to 8 and 30 empirically for most point clouds, respectively, with low and high densities. Note that these values work well for most point clouds but may require fine-tuning for some special cases. The factor μ is empirically set to 2. θ and $\sigma_{n}$ are set to 150° and 15°, respectively. The proposed approach is implemented using MATLAB MEX and run on Inter(R) Xeon(R) CPU E5-1620 @ 3.60 GHz with 8 GB RAM.

### 5.2. Processing Time

Table 1 provides the processing time of our approach, which is mainly affected by the number of neighbors (*N*) and the number of optimization iterations (iter). It is worth noting that the proposed approach converges in quite a few iterations and exhibits short processing time due to its parallel processing for all points.

### 5.3. Quantitative Results

The Chamfer measure, also known as Cloud-to-Cloud (C2C) distance, is computed to evaluate the denoising performance quantitatively. It reflects the matching distance between a denoised point cloud and the target one and is defined as the average distance between the denoised point cloud and the ground truth:

(13) $$\frac{1}{2}\{\frac{1}{N_{1}}\sum_{j=1}^{N_{1}}\min_{i}{∥p_{i}-{\hat{p}}_{j}∥}_{2}^{2}+\frac{1}{N_{2}}\sum_{i=1}^{N_{2}}\min_{j}{∥p_{i}-{\hat{p}}_{j}∥}_{2}^{2}\},$$

where the first term is the average distance between each processed point and its closest ground-truth point, and the second term is the one between each ground-truth point and its closest processed point.

Table 2 quantifies the performance of different approaches on representative point clouds based on Chamfer measure. The second row measures the noise levels of different point clouds.

Note that, as sculpture, armadillo and elephant models are raw scans without ground truth, whose noise levels cannot be measured, we assess the deviation of processed results from raw scans to quantify the changes introduced by the approaches. In this regard, the approaches that yield intermediate Chamfer distances—neither too small nor too large—produce the most favorable denoised models. Small distances indicate insufficient denoising, while large distances imply the erosion of important shape features.

In the case of the wedge model (Figure 6), EAR achieves the lowest Chamfer distance from the ground truth despite producing a gap along the edge (see Figure 6e). This is because EAR aims to evenly distribute points during smoothing, which generates the point cloud with closer overall distribution to the ground truth (see Figure 6a). PCN only slightly reduces noise and yields the largest Chamfer distance. APSS, RIMLS and LM show similar performance, while RIMLS performs marginally better. CLJNEPCF achieves the second-lowest Chamfer distance, largely due to its uniform point distribution akin to EAR, and outperforms APSS, RIMLS, PCN and LM, which is consistent with the visual comparisons (see Figure 6g). Our approach outperforms APSS, RIMLS, PCN and LM and accordingly delivers a better visual result (see Figure 6i). Note that, although our approach yields a larger Chamfer distance than CLJNEPCF, owing to CLJNEPCF’s ability to generate more evenly distributed points, it nevertheless produces a better shape visually.

In terms of the Fandisk model (Figure 7), PCN yields the largest Chamfer distance. Different from its performance on the wedge model, EAR has the second-largest error. LM performs slightly better than EAR. APSS, RIMLS and CLJNEPCF show similar and improved performance compared to the first three methods. Our approach achieves the best result.

For the twisted vase model (Figure 1), the quantitative evaluation exhibits a trend similar to that of the Fandisk model, with the exception that EAR produces the largest error. CLJNEPCF and our approach deliver the lowest errors.

As for the raw scan models (Figure 8, Figure 9 and Figure 10a), EAR results in the largest Chamfer distance as it tends to over-smooth the models. LM follows EAR, with the second-largest distance originating from both over-smoothing and over-sharpening issues that introduce gaps and holes visually. PCN achieves the overall lowest distance by making minimal changes to the original raw scan model. APSS, RIMLS, CLJNEPCF and our approach produce faithful results with similar and moderate distances.

### 5.4. Visual Results

#### 5.4.1. Synthetic Point Clouds

Figure 4, Figure 5 and Figure 6 show the denoising results for regular geometric shapes, and Figure 1 and Figure 7 show those for complex irregular geometric shapes.

Figure 4 shows the result of applying the proposed approach on a cubic corner, a representative shape that can test the edge-awareness of an approach. The surfaces are smoothed with both edge and vertex features well preserved.

In Figure 5, we test our approach upon a closed dodecahedron shape with different parameter settings. The parameters include the potential maximum angle in features θ, the smoothing coefficient λ, and the number of iterations iter, which is determined by $\beta_{max}$ and μ. (i) The comparison between Figure 5c,f shows the effect of θ on denoising. The result obtained with θ<108° in Figure 5c has slightly blurred edges, raised vertices and uneven faces due to improper latent group generation, while the one with θ>108° in Figure 5f has relatively smooth faces with sharp edges and vertices. Note that θ is employed to ascertain whether edges exist. A small value of θ can omit and thus over-smooth inherent soft features, while a large one can protect both sharp and soft features but probably misjudge some noise as features. Therefore, to protect features in most point clouds, a tradeoff can be made by setting a relatively large value. (ii) iter defines the number of iterations carried out to process the point cloud. The comparison between Figure 5d,f shows that a larger iter can generate a more compact point model with both clear boundaries and smooth faces. (iii) Figure 5e–h show the evolution of denoising results with increasing λ and reveal that a larger λ can better contribute to feature and face restoration. When λ≥0.1, the result starts to converge.

In Figure 6, the denoising results of the proposed approach on a wedge shape are compared with those of the baselines. APSS and RIMLS obtain similar results with over-smoothed and smeared edges. This aligns with the characteristics of MLS-based approaches that tend to blur sharp features. The wedge model processed by EAR consists of evenly distributed and denser points but includes a gap and distorted surfaces. This is because EAR employs local optimal projection with anisotropic weights to preserve features, but it has the drawback of producing gaps. PCN minimally denoises the point cloud and produces a relatively coarse model. CLJNEPCF produces a generally good shape despite small imperfections around edges. LM smooths faces and recovers the edge but slightly distorts the edge and yields gaps. LM utilizes $\ell_{0}$ optimization to smooth both normals and points under the assumption of global sparsity for noise. It can preserve features but is sensitive to noise levels, and its post-edge-recovery process often leads to gaps and holes. Our approach can restore flat faces and the edge, producing clear and straight boundaries. The results on both the cubic corner and the wedge demonstrate that our approach is capable of processing open surfaces.

Figure 1 shows the denoising result of our approach on a twisted vase point cloud containing irregular corners and twisted ridges with small edge angles (<30°). Figure 1c shows that both curved surfaces and sharp features (e.g., ridges and corners) are faithfully restored. Figure 1d further demonstrates, in detail, from the view of the top inside of the model, that clear edges are preserved.

Figure 7 compares the performances of different approaches on the Fandisk point cloud. APSS generates smooth surfaces but over-smooths sharp edges. RIMLS produces relatively smooth surfaces and preserves edges, but the areas along edges are slightly distorted. EAR obtains smoother surfaces than those of the first two approaches but slightly smears edges and leaves some corners not being well preserved. PCN reduces noise but makes minimal changes to the model, resulting in coarse surfaces. Despite the overall performance, CLJNEPCF leaves the model with an insufficiently smooth surface and slightly degraded edges and corners. LM yields smooth surfaces but slightly distorts edges and introduces gaps and holes on surfaces and edges. Our approach restores surfaces with features (i.e., edges and corners) well preserved. The mesh built on the result of our approach shows the clear contour of the Fandisk model. Note that the ball-pivoting algorithm is used for mesh reconstruction and the pseudoinverse quadric fitting method is applied to generate colors.

#### 5.4.2. Real-World Raw Scans

In Figure 8, Figure 9 and Figure 10, the experiments are conducted on real-world raw scans characterized by noise and nonuniform point distribution.

The bronze sculpture shown in Figure 8 is typically composed of plenty of sharp features and curved surfaces. APSS generates a smooth model but tends to over-smooth edge details, especially in the areas of eyes, eyebrows, nose and mouth. RIMLS smooths the surface with edge features well preserved. The model processed by EAR retains some prominent edge features (e.g., nose and eyes) but exhibits blurring in smaller ones (e.g., mouth). PCN produces a smoothed result with edge features, showing that it can handle point clouds with low-level noise. CLJNEPCF introduces extraneous geometric features that are absent from the ground-truth model, particularly in regions such as the glabella, forehead, nasal alae, and periocular areas. LM over-smooths some features (e.g., nose and eyebrows), introduces gaps and holes near some edges, and over-sharpens some small edge features (e.g., ears). Our approach achieves a smooth model while maintaining clear edge features. Figure 8i shows the result of the mesh surface reconstruction based on the point cloud generated by our approach.

The armadillo model in Figure 9 is typically characterized by soft features (e.g., the flower textures and wrinkles on the body, the eye, etc.) and sharp features (e.g., the ear, the mouth, the feet, etc.). While all the approaches yield similar smooth results, they differ in preserving details in various parts, such as the mouth, flower textures, ear, etc. APSS and EAR slightly over-smooth features such as the mouth and flower textures, while RIMLS and our approach preserve most of the features. As the armadillo raw scan presents a similar low-level noise to the bronze sculpture model, PCN produces a well-smoothed result while maintaining key features. CLJNEPCF over-smooths some important features such as flower textures and eyes, fails to protect the ear tip structure, and introduces some extraneous geometric features (e.g., the circular artifact around the neck; textures on the body surface) that are absent from the ground-truth model. LM over-sharpens some edge features around the mouth and ear and introduces some gaps and holes on the ear and feet.

Figure 10 shows additional denoising results of the proposed approach on different point clouds, including the elephant, iron vise and shutter models. In Figure 10a, the features of the elephant, including the nose, eye, feet and skin, are well restored. Figure 10b and Figure 10c further demonstrate that our approach can smooth surfaces and preserve geometric details of sharp features (e.g., edges and corners).

## 6. Conclusions

In this paper, we revisit sparse optimization-based point cloud denoising and contribute in the following aspects. First, we propose a latent group and feature identification approach to characterize local structures of point clouds. Then, we introduce a group-level sparsity-based approximate deviation that provides appropriate distance measures for both feature and facet points. Based on that, we build a structure-aware $\ell_{0}$ optimization model that jointly processes different types of points, preventing structural biases and inconsistencies. We further obtain a linear approximate solution and present a parallel denoising algorithm. The experimental results demonstrate that our approach can outperform baselines.

## Figures and Tables

**Table 1:** Timing for our approach on different point sets.

Models	Points	*N*	iter	Time (s)
Twisted vase	88,518	30	6	10.1
Cube corner	30,301	30	8	4.1
Dodecahedron	72,719	30	4	3.7
Wedge	22,801	30	8	2.9
Fandisk	88,161	30	8	13.7
Sculpture	98,507	8	4	2.8
Armadillo	99,416	8	4	2.9
Elephant	150,005	8	4	4.3
Iron vise	161,004	30	8	22.1
Shutter	291,220	30	8	41.9

**Table 2:** Denoising performance with Chamfer measure ($\times 10^{-6}$).

	Wedge	Fandisk	Twi.Vase	Sculp.	Armadi.	Elephant
Noisy	61.553	33.520	48.285	-	-	-
APSS [7]	36.530	4.979	11.126	0.705	2.193	1.551
RIMLS [8]	35.706	6.656	10.684	0.310	0.783	1.558
EAR [13]	8.170	14.307	33.086	134.820	47.991	54.610
PCN [17]	53.777	19.975	19.341	0.051	0.239	2.586
CLJNEPCF [21]	19.057	4.713	5.045	1.446	1.745	2.145
LM [23]	35.915	10.995	13.167	11.560	7.600	42.447
Ours	30.224	0.462	5.268	0.918	1.100	2.163

## Data Availability

The data presented in this study are available on request from the corresponding author due to privacy.

## Appendix A. Solution to Sub-Problem I

We present here the derivation of the solution to Sub-Problem I in Equation (6). As the term $∥\omega_{i}^{m}{∥}_{0}$ in Equation (6) is binary, we rewrite Equation (6) as Equation (A1) by considering that either $\omega_{i}^{m}=0$ or $\omega_{i}^{m}\neq 0$:

(A1) $$\min\left\{\min_{\omega_{i}^{m}=0}\beta {|\nabla P_{i}^{m}|}^{2},\;\;\min_{\omega_{i}^{m}\neq 0}\beta {\left|\nabla P_{i}^{m}-\omega_{i}^{m}\right|}^{2}+\lambda \right\}.$$

As the second term in Equation (A1) can be minimized only if $\omega_{i}^{m}=\nabla P_{i}^{m}$, the problem can further be written as:

(A2) $$\min\left\{\min_{\omega_{i}^{m}=0}{\beta |\nabla P_{i}^{m}|}^{2},\;\;\min_{\omega_{i}^{m}=\nabla P_{i}^{m}}\lambda \right\}.$$

Through comparing two terms in Equation (A2), we can obtain the solution to $\omega_{i}^{m}$:

(A3) $$\omega_{i}^{m}=\left\{ \begin{array}{cc} 0, & \mathrm{if}\;\lambda /\beta >{|\nabla P_{i}^{m}|}^{2},\\ \nabla P_{i}^{m}, & \mathrm{otherwise}, \end{array}\right.\;\forall i\in I,\forall m\in M_{i}.$$

## Appendix B. Solution to Sub-Problem II

To derive the solution to Sub-Problem II in Equation (9), we rewrite the problem by defining $f(p_{i})$.

(A4) $$f\left(p_{i}\right)\;=\;{∥p_{i}-{\tilde{p}}_{i}∥}_{2}^{2}+\beta \;\;\sum_{m\in M_{i}}\;\;\;{\left(\frac{1}{|G_{i}^{m}|}\;\sum_{j\in G_{i}^{m}}\;n_{j}^{T}\left(p_{i}-p_{j}^{k}\right)-\omega_{i}^{m}\right)}^{2}.$$

To obtain $p_{i}$ that minimizes $f(p_{i})$, we compute the equation $\frac{\partial f(p_{i})}{\partial p_{i}}=0$, whose expanded form is shown in Equation (A5):

(A5) $$\begin{array}{cc} \; & p_{i}-{\tilde{p}}_{i}+\beta \sum_{m\in M_{i}}\Omega_{i}^{m}{\bar{n}}_{i}^{m}=0, \end{array}$$

(A6) $$\begin{array}{cc} \; & \Omega_{i}^{m}=\frac{1}{|G_{i}^{m}|}\sum_{j\in G_{i}^{m}}n_{j}^{T}\left(p_{i}-p_{j}^{k}\right)-\omega_{i}^{m}, \end{array}$$

(A7) $$\begin{array}{cc} \; & {\bar{n}}_{i}^{m}=\frac{1}{|G_{i}^{m}|}\sum_{j\in G_{i}^{m}}n_{j}. \end{array}$$

Equation (A6) can be further expanded and rewritten as:

(A8) $$\Omega_{i}^{m}=\frac{1}{|G_{i}^{m}|}\sum_{j\in G_{i}^{m}}n_{j}^{T}p_{i}-\frac{1}{|G_{i}^{m}|}\sum_{j\in G_{i}^{m}}n_{j}^{T}p_{j}^{k}-\omega_{i}^{m}.$$

Substituting Equation (A8) into Equation (A5), we obtain:

(A9) $$\begin{array}{cc} \; & p_{i}-{\tilde{p}}_{i}+\Psi_{i}p_{i}-\Phi_{i}=0, \end{array}$$

(A10) $$\begin{array}{cc} \; & \Psi_{i}=\beta \sum_{m\in M_{i}}{\bar{n}}_{i}^{m}{\left({\bar{n}}_{i}^{m}\right)}^{T}, \end{array}$$

(A11) $$\begin{array}{cc} \; & \Phi_{i}=\beta \sum_{m\in M_{i}}\left(\omega_{i}^{m}+\frac{1}{|G_{i}^{m}|}\sum_{j\in G_{i}^{m}}n_{j}^{T}p_{j}^{k}\right){\bar{n}}_{i}^{m}. \end{array}$$

Therefore, $p_{i}$ can be computed as:

(A12) $$p_{i}={\left(I+\Psi_{i}\right)}^{-1}\left({\tilde{p}}_{i}+\Phi_{i}\right).$$

Finally, substituting Equations (A10) and (A11) into Equation (A12), we can obtain the solution below.

(A13) $$\begin{array}{cc} \; & p_{i}^{k+1}=U_{i}^{-1}v_{i},\;\forall i\in I, \end{array}$$

(A14) $$\begin{array}{cc} \; & U_{i}=I+\beta \sum_{m\in M_{i}}{\bar{n}}_{i}^{m}{\left({\bar{n}}_{i}^{m}\right)}^{T}, \end{array}$$

(A15) $$\begin{array}{cc} \; & v_{i}={\tilde{p}}_{i}+\beta \sum_{m\in M_{i}}\left(\omega_{i}^{m}+\frac{1}{|G_{i}^{m}|}\sum_{j\in G_{i}^{m}}n_{j}^{T}p_{j}^{k}\right){\bar{n}}_{i}^{m}. \end{array}$$

The position of point $p_{i}$ can be updated based on Equation (A13).
